# Drawing on Adolescent Psychology to Achieve Tobacco-Free Generations

**DOI:** 10.3389/phrs.2022.1604321

**Published:** 2022-08-04

**Authors:** Jon Berrick

**Affiliations:** Yale-NUS College, Singapore, Singapore

**Keywords:** adolescent psychology, motorcycle helmets, tobacco-free generation, under-age laws, tobacco endgame

## Abstract

**Background:** In 2021, the European Union called for creation of a “tobacco-free generation.” We consider the means to this end. The persistence of youthful noncompliance with current minimum age laws (leading to widespread subsequent addiction, morbidity and mortality) raises questions whether such laws are truly aligned with adolescent psychology.

**Evidence:** The ubiquity of minimum-age laws limits direct evidence of their effectiveness, so we seek indirect evidence. Qualitative findings originally intended for tobacco manufacturers indicate counterproductive aspects of minimum-age laws. Further evidence about adolescent reactions is provided by a recent review and meta-analysis of greater youth defiance of under-age laws than whole-of-life laws in the domain of motorcycle helmets.

**Policy Options and Recommendations:** As an alternative to minimum-age laws, we consider the Tobacco-Free Generation proposal (TFG), which phases out sales on an age cohort basis and has recently gained prominence.

**Conclusion:** The Tobacco-Free Generation proposal (TFG) seems well aligned with adolescent psychology, and is therefore especially worthy of attention. It has recently been introduced or endorsed by a number of jurisdictions, both local and national.

## Background

The European Union (EU) is proposing a “‘tobacco-free generation’ where less than 5% of the population uses tobacco by 2040, compared to around 25% today” [1, p.1]. The EU call is prompted by the knowledge that “cigarettes kill more people each year than AIDS, heroin, crack, cocaine, alcohol, car accidents, fire, and murder combined” [2] (and more than Covid-19 since 2020), and follows the theme for the 17th World Conference on Tobacco or Health (Cape Town, 2018): “Uniting the World for a Tobacco Free Generation.” The details of how to achieve this admirable goal, sometimes described as “elimination” or an “endgame”, remain to be fleshed out. Recent limited progress [3] highlights the need for fresh thinking.

Because tobacco consumption is associated with strong dependence [4, p.571] (and early hopes for electronic nicotine delivery systems as a remedy have not been supported by the most recent evidence [5]), it can be deduced that the greatest progress towards the elimination goal will be achieved by minimising initial adoption of tobacco consumption. In turn, tobacco take-up is recognised to occur predominately among teenagers [4, p.573]. To address this pattern of behavior, it is important to understand teens’ motivations. Consequently, we investigate the role that adolescent psychology can play in the quest for laws that help prevent tobacco initiation among youth.

A particular challenge is the stock usage of minimum-age laws ostensibly aimed at prevention for this age group; this leads to a shortage of empirical studies of their efficacy [6]. To test the wisdom of this convention, we are thereby obliged to be more indirect. Thus, we draw upon tobacco industry documents for their qualitative research findings on teenagers’ attitudes to these measures. Moreover, since tobacco is not the only domain of public health and safety where age-restricted laws apply, one can look for lessons offered by experiences elsewhere in order to gauge the impact of such laws. This bears on the question of how much the expressive power (messaging) of legislation can influence adolescent motivation and thence behavior. A recently published systematic review and meta-analysis on youth reaction to US motorcycle helmet laws [7] provides novel insight into relevant adolescent psychology, and draws attention to the role that widespread minimum-age laws may play in the initiation of young people.

Our aim is to investigate relevance and implications of these adolescent psychology findings for minimum-age laws in current use, focussing on how they can inform tobacco policy aimed at achieving tobacco-free generation goals. We review evidence and then discuss tobacco policy implications, concentrating on ultimate elimination rather than reduction of extent or impact.

## Evidence

### Direct Evidence on Under-Age Tobacco Laws

Unfortunately, the ubiquity of age-specific tobacco laws discourages questions about their effectiveness. Instead, debate usually centres on choice of a “right” age (such as the recent Tobacco21 debate in the US relating to raising the minimum sale age to 21 years). Often, this is framed in terms of responsibility: a conventional argument is that persons deemed responsible enough to vote in elections or enlist in a country’s military forces are sufficiently responsible to take part in any other “adult” activity. But what if, because of health and safety concerns, it is preferable that adults *not* take part in the activity, even if, for historical reasons, some currently do so? Tobacco, leading annually to more than seven million premature deaths globally, is the stand-out example here.

Regarding efficacy of age-restrictive tobacco laws, an intervention-control pre-post study of 19 EU countries found that laws prohibiting the sales of tobacco to minors there “do not appear to be associated with a reduction in adolescent smoking rates” [8, p.320], echoing a 1999 literature review which concluded that “setting an age limit for buying cigarettes has little impact” [9, p.596]. A recent South Korean study attributed minimum-age laws’ limitations to a rite-of-passage effect [10]. Such findings are consistent with an experimental psychology study suggesting that age-restricted laws (specifically for tobacco) have the effect of creating young adult role models for adolescents in a way that does not otherwise occur [11].

A recent systematic review based on over 3000 articles found limited evidence to understand how an age-of-sale ban affected youth smoking behavior, noting the absence of studies evaluating the effects of the ban as distinct from other policies enacted simultaneously [6]. We are thus obliged to consider alternative approaches to inference of effectiveness of minimum-age laws—provided by the tobacco industry’s own qualitative research findings, and by the experience of departures from age-specific laws in other domains of public health and safety.

### Tobacco Industry Strategy and Findings

One of the most telling critiques of under-age laws is their advocacy by corporations whose continuing need for new customers can be met only when controls are ineffectual. Minimum-age laws are advocated, sought, and stoutly defended by the tobacco industry. For example, from the Altria CEO: “there should be minimum age laws. We led the effort on that, making sure there were minimum age laws in place everywhere and that they were vigorously enforced” [12]. The logic for the tobacco industry to take such a stand is quite straightforward.

• Initiation occurs overwhelmingly among adolescents.
• The product is highly addictive, bringing potentially lifelong customers.
• Therefore, it is important to market to adolescents.

The next step is the optimization of this marketing. The Masters Settlement Agreement Truth document repository of internal tobacco industry documents affords insight into the tobacco industry’s approach. It is clear that, through its internal market research (for example, through focus-groups of teenagers), the industry has long been aware of the key role of adolescent psychology (revealingly, several decades before its emergence in the tobacco control literature), and in particular its reaction to age-restricted laws, exemplified by the following aspects.

#### Mixed Messaging

A difficulty for the industry is the evidence highlighted by public health workers (but characteristically downplayed by the industry) of health dangers of smoking. However, minimum-age laws provide the industry with an opportunity to muddy the waters, by touting tobacco as a “legal product.” If indeed the product is safe enough to be available to adults—even those just above the minimum age—then the laws are open to the inference that their real aim is youth control rather than tobacco control [13].

#### Assertion of Independence

Given this reframing, reactance theory (relating to resentment of imposed behavioral constraints) [14] predicts that youth legally excluded from the product will find it more desirable. Thus, internal qualitative research for Imperial Tobacco in Canada asserted that “cigarettes. . . are associated with adulthood and at the same time adults seek to deny them to the young. By deliberately flaunting out this denial, the adolescent proclaims his break with childhood, at least to his peers” [15, p.6/110]. Market research for Brown & Williamson claimed that “This illicit pleasure will lose its illicitness once they grow older” [16, p.7/9].

#### Illusion of Maturity

Youthful desire of an image of maturity, as perceived both by self and others, is exploited in media by product placement [17], often by just-over-minimum-age role models. The Imperial Tobacco research stressed that in the presence of under-age laws, cigarettes become “a badge of coming of age, a symbol of the onset of maturity” [15, p.51/110]. A planning memorandum for R.J. Reynolds argued that “The fragile, developing self-image of the young person needs all of the support and enhancement it can get. Smoking may appear to enhance that self-image in a variety of ways … This self-image enhancement effect has traditionally been a strong promotional theme for cigarette brands and should continue to be emphasized.” [18, p.8/12] Brown & Williamson’s research labelled the cigarette “the entrance ticket to the hall of the adult society” [16, p.8/9].

### Evidence From Alternative Domains

Two domains where evidence is available on the impact of alternatives to minimum-age laws are those of opium-smoking (discussed in [19]) and motorcycle helmets, on which we now focus.

#### Motorcycle Helmets

A recently published paper [7] considers the varied US state regimes governing adolescent motorcycle helmet usage (some states have shifted between mandating all motorcyclists to be helmeted and requiring this only of those under age 18 or 21), to investigate the effect on adolescent noncompliance of an age-specific law instead of a non-age-defined, whole-of-life law. Since the relationship to tobacco control is less obvious than for opium-smoking, we emulate Table 2 of Schroeder [20] (comparing characteristics of tobacco use and obesity, in order to determine which tools of tobacco control might be applicable to combating obesity). In Table 1 above, we itemize a number of factors that may influence adolescent defiance of laws mandating motorcycle helmet usage or abstention from tobacco. Important similarities include the messages of youth control conveyed to adolescents by age-restrictive laws and the related scope for peer pressure and maturity-signalling defiance. Differences arise from tobacco’s being heavily marketed (by social media or screen product placement even where direct advertising is banned).

**TABLE 1:** Determinants of youths’ avoiding motorcycle helmets and taking up tobacco: similarities and differences (Singapore, 2021).

Influential factor	Helmet	Tobacco Uptake
	Avoidance	
Public warnings of danger	Present	Present
Visibility of imminent danger	Absent	Absent
Scope for self-exemption from danger warnings	Present	Present
Declarative message of age-specific or generic law	Present	Present
Maturity symbolism of defiance	Present	Present
Peer pressure	Present	Present
Libertarian rhetoric	Present	Present
Threat of apprehension	Present	Present
Possibility of collateral social harm (load on hospitals, & c.)	Present	Present
Convenience/comfort	Present	Absent
Financial incentive	Present	Absent
Marketing pressure	Absent	Present
Addictiveness	Absent	Present

Libertarian and rider organization lobbyists associated with motorcycling share with tobacco marketers denial of well-established science on health/safety benefits of regulation [21–23]. The addictiveness of nicotine of course has no counterpart for helmets. However, inasmuch as our focus is on motivation for youth initiation of tobacco consumption, the issue of addictiveness is less pertinent.

The meta-analysis of [7] finds that about two-thirds of youth defiance of an age-restricted helmet law disappears when replaced by a universal law, a law that for each adolescent has whole-of-lifetime rather than limited duration applicability, with evidence that “a large part of the greater compliance with universal laws is due to their conveying a more convincing message that helmets afford protection against injury” [7, p.166]. Studies of youths’ reactions point to the cogency of the messaging that universal laws are intended for protective benefit whereas age-specific laws may be perceived by adolescents as signalling authorities’ desire for youth control. It is noteworthy that beneficial changes in youth behavior occurred even in the period between the announcement of a universal law and its implementation.

## Policy Options and Recommendations

The findings above on the efficacy of universal helmet laws for increasing adolescent compliance with such laws suggest that the *Tobacco-Free Generation proposal (TFG)* [19, 24] that has recently been adopted by several jurisdictions is likely to reduce youth tobacco initiation. A brief description is as follows.

While addictiveness of nicotine precludes the sudden introduction of a universal law banning sales (“prohibition”) [25], it is possible to avoid this hazard by a process of grandfathering. TFG provides a “generational firebreak” [26] by adopting a cohort-based approach. (While the term “a tobacco-free generation”, as a descriptor for a low-tobacco-prevalence cohort, met occasional prior use [27], it seems to have become more common following attention paid to the “Tobacco-Free Generation proposal”, initially so named at the Workshop on End Game Strategies in Tobacco Control, University of Michigan, June 2012 [23].) TFG forbids sales to those born after a certain fixed date—a steadily increasing proportion of the population. (In Europe, a cut-off date of 1 January 2010, chosen to leave existing smokers unaffected, would leave minimum age of 18 laws in force for another 6 years before becoming superfluous.) It is important to appreciate that from the perspective of the key actor—the individual adolescent—the initiative functions like a universal motorcycle helmet law in that it has whole-of-life impact. Like the historical approach to opium-smoking referenced above, it grandfathers customers, while (for ease of enforcement) concentrating its policing attention on vendors—the purveyors of the harmful substance—rather than on their customers. In operation, as its smoking cohorts age away from adolescence and thus provide a decreasing role model for new generations, it may be expected to progressively normalize so as to become effectively irreversible (cf smoke-free public transport).

In recent times, TFG was successfully introduced in Balanga City, Philippines from 2016 (using 1 January 2000 as the cut-off date) [28] though later subjected to tobacco industry litigation (for alleged nonalignment with national laws) [29]. In the US, it has been adopted in Brookline, Massachusetts (again with 2000 cut-off) [30–32]. It is administratively simple: a birth date on an ID is easier for a vendor to check than a computation of age. The policy is most suited to jurisdictions where tobacco retailers are licenced, facilitating monitoring and enforcement [33], and needs to be supported by vigorous publicity to reinforce its health messaging.

A common argument against TFG is that prohibition of a substance or activity makes it more desirable. Tolstoy’s observation that “There are no conditions of life to which a man cannot get accustomed, especially if he sees them accepted by everyone around him.” [34, pt.7, ch.13] highlights that more nuance is needed. The evidence of very many well-accepted laws involving some kind of prohibition (virtually everywhere it is prohibited to drive on the “wrong” side of the road; numerous consumer products respect prohibitions of certain ingredients; in the US, the Food & Drug Administration each year prohibits hundreds of potentially marketable substances) is that prohibition can be successful if the public is persuaded that it serves their interest—as, for example, TFG legislation sending the message that there is no safe age for tobacco consumption.

Perhaps the main argument against TFG is that it takes a long time (albeit arguably surer than competing strategies [35]) to reach eradication, although the evidence of the recent systematic review and meta-analysis on youth reaction to US motorcycle helmet laws [7] signals that its messaging holds the possibility of social norm changes bringing about faster gains.

Experience in the Australian state of Tasmania (where a proposal for TFG was made by an independent legislator in 2014 but lapsed when an election was called) as well as Philippines and Massachusetts cited above shows that TFG passes the tobacco control “scream test” [36]. It has roused the tobacco industry to label it “prohibition”, to encourage libertarian objections, and to instigate vendor protests over potential loss of livelihood (despite such loss needing ultimately to be material only for dedicated tobacconists— for other vendors, money not diverted to tobacco products remains available for customers to spend on other goods and services).

Nevertheless, opinion polling suggests that TFG attracts widespread public approval [25, 37, 38]. It has also been endorsed by numerous public health bodies including the British and Norwegian Medical Associations and the 16th World Conference on Tobacco Or Health [36, 39–41].

More recently, New Zealand became the first nation to announce its intention to legislate TFG, in 2022, using a cut-off date likely to be 2009 [42–45]. Subsequently, health ministries in Malaysia and Denmark have also announced plans to introduce TFG in 2022 (indicating cut-off dates of 2005 and 2010 respectively) [46, 47]. Responding to public urging [48], Singapore’s health minister considers TFG “an attractive policy”, and “will study how New Zealand implements the ban, its effectiveness and how their experience could be applicable to Singapore” [49]. Thus, this is currently an active focus of international policy attention.

## Conclusion

The phrase “tobacco-free generation” has now gained currency within the EU [1]. Evidence both directly regarding tobacco and from a just-published review and meta-analysis of youth reaction to US under-age motorcycle helmet laws [7] indicates that minimum-age laws are an impediment to achieving this goal. This provides further support for the Tobacco-Free Generation policy (TFG)—confining vendors’ customers to those born prior to a suitable cut-off date. It is worthy of consideration for jurisdictions seeking to make tobacco-free generations a reality.
